# The Past, Present, and Future of *Kingella kingae* Detection in Pediatric Osteoarthritis

**DOI:** 10.3390/diagnostics12122932

**Published:** 2022-11-24

**Authors:** Pablo Yagupsky

**Affiliations:** Clinical Microbiology Laboratory, Soroka University Medical Center, Ben-Gurion University of the Negev, Beer Sheva 8410500, Israel; pyagupsky@gmail.com; Tel.: +972-50-626-4359

**Keywords:** *Kingella kingae*, children, osteoarthritis, diagnosis, culture, nucleic acid amplification tests, metagenomic next-generation sequencing

## Abstract

As a result of the increasing use of improved detection methods, *Kingella kingae*, a Gram-negative component of the pediatric oropharyngeal microbiota, is increasingly appreciated as the prime etiology of septic arthritis, osteomyelitis, and spondylodiscitis in children aged 6 to 48 months. The medical literature was reviewed to summarize the laboratory methods required for detecting the organism. *Kingella kingae* is notoriously fastidious, and seeding skeletal system samples onto solid culture media usually fails to isolate it. Inoculation of synovial fluid aspirates and bone exudates into blood culture vials enhances *Kingella kingae* recovery by diluting detrimental factors in the specimen. The detection of the species has been further improved by nucleic acid amplification tests, especially by using species-specific primers targeting *Kingella kingae*’s *rtxA*, *groEL,* and *mdh* genes in a real-time PCR platform. Although novel metagenomic next-generation technology performed in the patient’s plasma sample (liquid biopsy) has not yet reached its full potential, improvements in the sensitivity and specificity of the method will probably make this approach the primary means of diagnosing *Kingella kingae* infections in the future.

## 1. Introduction

Infections of joints, bones and intervertebral discs in children are medical emergencies that require early diagnosis and proper treatment to avoid severe morbidity and prevent permanent orthopedic disabilities [1,2]. Based on traditional microbiological methods such as Gram stains and cultures on solid media, *Staphylococcus aureus* was historically considered the most common etiology of skeletal system infections in children of all ages [3]. In the 1960s, the routine introduction of chocolate-agar plates for seeding synovial fluid aspirates revealed the role of *Haemophilus influenzae* type b as the predominant agent of septic arthritis in young children, indicating that detection of some microorganisms requires specific growth conditions that need to be addressed by the Clinical Microbiology Laboratory (CML) [4]. However, even when agar-chocolate medium are employed and specimens are obtained prior to antibiotic therapy, cultures have a low yield, and 1/3 to 2/3 cases of suppurative arthritis, osteomyelitis, and spondylodiscitis remain microbiologically unproven [5,6].

In addition to their poor sensitivity, cultures take 1–5 days to isolate and identify bacteria and 1–3 days for antimicrobial susceptibility test results. Because of the lack of timely laboratory information, broad antimicrobial therapy is usually administered to cover the unknown agent of the infection. Failure to detect a bacterial pathogen could result from misdiagnosing viral or reactive arthritis and medical conditions that mimic osteoarticular infections such as rheumatologic or metabolic disorders, trauma, or malignancy [7,8,9]. Yet, the possibility that a negative culture could be caused by microorganisms that traditional laboratory techniques cannot isolate should also be considered. 

## 2. *Kingella kingae*: An Elusive Pediatric Pathogen

For most of the three decades following its first description, *Kingella kingae* was considered an exceptional agent of human disease, infrequently recovered from patients with skeletal system infections and bacterial endocarditis [10]. In 1988, it was reported that the inoculation of joint fluid aspirates into blood culture vials (BCVs) significantly increased the isolation of the bacterium, revealing that *Kingella kingae* is the prime etiology of septic arthritis and osteomyelitis in children aged 6–48 months [11]. More recently, nucleic acid amplification tests have further improved the detection of this fastidious organism and reduced the fraction of culture-negative osteoarthritis in children [12]. *Kingella kingae* is carried as part of the oropharyngeal microbiota in early childhood [13]. Facilitated by a potent RTX (repeats in toxin) pore-forming toxin and viral upper respiratory infections, the bacterium breaches the pharyngeal epithelium and enters the bloodstream, from which it disseminates to the skeletal system and the endocardial tissues [13].

The clinical picture of *Kingella kingae*’s joint and bone infections is characterized by a normal or slightly elevated body temperature and unimpressive acute-phase reactant levels, resembling transient synovitis [14]. *Kingella kingae* is intrinsically resistant to clindamycin and vancomycin and exhibits a high minimal inhibitory concentration (MIC) of isoxazole penicillins such as cloxacillin, which are empirically administered to children with suspected skeletal system infections, pending culture and antibiotic susceptibility results [15].

Due to the fastidious features of *Kingella kingae*, the organism is frequently missed by the CML, and pediatricians are not always aware of the special detection methods required to detect this difficult-to-culture pathogen. The present review aims to summarize the current techniques for diagnosing *Kingella kingae* infections in children, and their uses, advantages, and limitations, to improve the management of pediatric skeletal system infections.

## 3. *Kingella kingae* Identification

*Kingella kingae* exhibits a distinctive morphology, consisting of pairs or short chains of plump, Gram-negative coccobacilli with tapered ends [13]. The organism grows as small grey β-hemolytic colonies that produce marked pitting on the agar surface. All strains show facultative anaerobic growth and develop on blood-agar and chocolate-agar plates, but not on MacConkey agar. *Kingella kingae* is non-motile and displays positive oxidase and negative catalase urease and indol reactions. *Kingella kingae* produces acid from glucose and maltose but not from other carbohydrates and exhibits alkaline and acid phosphatase activity [13]. Traditionally, the CML identification of the bacterium was accomplished with manual biochemical tests, the commercial API NH card, and Vitek 2 instrument systems. In recent years, matrix-assisted laser desorption ionization-time of flight mass spectrometry (MALDI-TOF) and 16S rRNA gene sequencing have been added to the speciation tools [13].

## 4. Detection

### 4.1. Gram Stain

The traditional Gram stain has conserved its diagnostic relevance in skeletal system infections because it combines technical simplicity, inexpensive equipment, and a rapid (a few minutes’) turnaround time. Thus, a Gram stain should be routinely performed in synovial fluid aspirates, exudates, and ground skeletal tissues to confirm the diagnosis, suggest the presence of a given bacterial species, and guide the initial antibiotic coverage. Whereas the Gram stain examination of the joint fluid aspirate reveals the presence of *Staphylococcus aureus* in 75% of patients with arthritis, the success rate drops to less than half in those infected with Gram-negative organisms [16]. Since as many as 10^5^ organisms/mL are required to obtain a positive Gram stain result in human body fluids, a negative synovial fluid examination does not necessarily imply a sterile specimen [17]. False-negative results rates ranging from 25% to 50% compared to cultures have been reported [16]. In *Kingella kingae*-infected patients, the Gram stain of joint and bone specimens is rarely positive, probably because of the low bacterial concentration (median: 15 colony forming units (CFUs) per mL), and the difficulty in distinguishing the few Gram-negative coccobacilli against the pink-stained fibrin background [18,19,20,21].

### 4.2. Culture Detection of Kingella kingae

#### 4.2.1. Blood Cultures

Since *Kingella kingae*’s invasion of joints and bones is preceded by the hematogenous dissemination of the organism, blood cultures appear as a suitable tool for the bacteriological diagnosis [13]. Whereas blood cultures identify the agents of osteoarthritis caused by pyogenic bacteria in 31% of cases, recovery of *Kingella kingae* in blood cultures from children with joint or bone infections is uncommon [22]. Employing Bactec (Becton Dickinson, Cockeysville, MD, USA) BCVs, *Kingella kingae* was isolated in 81 Israeli children with culture-proven osteoarthritis, in 74 (91.4%) children from joint or bone specimens, and in only 6 (7.4%) from the blood and both the synovial fluid aspirate and a blood culture in a single patient (1.2%). These results indicate the bacteremic phase is transient and short-lived and, by the time signs of arthritis or osteomyelitis develop, the bacterium has been already eradicated from the bloodstream [13]. Despite their low sensitivity, blood cultures should always be obtained when skeletal system infections are suspected because they are easy to obtain and repeat and do not require the patient’s sedation. Due to the mild clinical presentation of *Kingella kingae* disease, blood cultures are indicated, disregarding the height of the body temperature, lack of leukocytosis, or normal acute-phase reactant levels [13,20].

#### 4.2.2. Synovial Fluid Aspirates

Cultures of joint fluid aspirates and bone biopsy samples in solid media frequently fail to recover *Kingella kingae* because the growth of the bacterium is inhibited by the presence of leukocytes, complement, antibodies, and eventual antibiotics in the sample [11,23,24]. The sensitivity of these cultures can be significantly improved by inoculating them into aerobic BCVs from the automated Bactec [11,25] and BacT/Alert systems (Organon Teknika Corporation, Durham, NC, USA) [26,27,28,29], the manual Hémoline DUO (bioMérieux, Marcy-l’étoile, France) [30,31] and Isolator 1.5 Microbial Tube (Wampole Laboratories, Cranbury, NJ, USA) [32] systems, and in-house-made liquid medium [33]. In a pioneering study, *Kingella kingae* was recovered in eleven joint fluid aspirates seeded into pediatric aerobic Bactec BCVs. In contrast, simultaneous conventional cultures on solid media isolated the bacterium in only two cases [11]. Subcultures of positive BCVs’ broth onto blood-agar and chocolate-agar plates grew the organism in all cases, showing that conventional bacteriological media support *Kingella kingae*’s growth demands. It is postulated that inoculating the tiny synovial fluid aspirates drawn from a small joint of a young child into a large volume of liquid medium dilutes putative inhibitory factors, facilitating detection [11]. Usually, the automated blood culture systems disclose the presence of *Kingella kingae* in the BCVs within four days, and prolonged incubation over the customary five-to-seven-day period does not further increase the isolation rate [34]. The consistent inoculation of synovial fluid aspirates drawn from children aged 6–48 months old into Bactec [35] or BacT/Alert [36] BCVs revealed that *Kingella kingae* was the prime pathogen of septic arthritis in this population segment and was isolated in one-half of those with bacteriologically proven infections.

The question of which blood culture systems and BCV media are preferable for recovering *Kingella kingae* from joint and bone infections remains unsolved. Since *Kingella kingae* osteoarthritis occurs mainly in children, the clinical experience is limited to the use of the pediatric formulations of the two most common blood culture systems, Bactec (Peds Plus BCVs) and BacT/Alert (Pedi-bacT BCVs) [13]. A study employing simulated synovial fluid cultures suggested that the BacT/Alert Aerobic and BacT/Alert Pedi-bacT BCVs outperformed the Bactec Peds Plus vials in terms of sensitivity and time-to-detection [37]. However, prospective volume-controlled comparative studies employing synovial fluid aspirates from actual patients are necessary to provide a definitive answer. In addition to the insurmountable difficulties of enrolling a sufficient number of pediatric patients with culture-proven *Kingella kingae* infections, CMLs employ a single automated blood culture system. Therefore, a proper comparison will probably never be performed. In any case, choosing a blood culture system is a costly and strategic decision with broad and long-lasting implications. The choice should be made based on a variety of professional and economic considerations and not only on the ability of the system to isolate a particular bacterial species.

#### 4.2.3. Bone Exudates and Specimens Obtained by Biopsy

The culture of bone exudates entails technical difficulties because of the high density of the material obtained by surgical debridement. If a lytic lesion is present, the exudate should be aspirated and inoculated onto a BCV [11]. Multiple specimens should be obtained from several sites and depths to improve the chances of detecting the causative organism. Due to the fastidious nature of *Kingella kingae*, the collected samples should be immediately plated on solid media and inoculated onto BCVs in the surgical theatre. The importance of seeding the exudates on culture media without delay was demonstrated in a small study in which intraoperative plating enabled *Kingella kingae*’s detection in five of six cases of osteomyelitis, compared to a single successful isolation when specimens were later transferred to the CML and processed according to routine methods [38].

### 4.3. Detection by Polymerase Chain Reaction (PCR)

In recent years, the development and implementation of nucleic acid amplification tests (NAATs) have revolutionized the field of infectious diseases. These culture-independent molecular techniques enable the detection of the etiologic agent of the infection within a few hours instead of days, disregarding the cell viability, prior antibiotic exposure, or fastidious growth. The use of molecular methods has expanded the list of human pathogens and revealed the presence of antibiotic-resistance-associated genes, enabling targeted antimicrobial therapy and improved laboratory safety [39]. The PCR amplification method can be tailored to detect a specific microbial species, a wide range of pathogens, or the most common agents causing a clinical syndrome (e.g., central nervous system infections, septic arthritis, etc.) in a single multiplexed assay [40].

The original approach consisted of extracting DNA from clinical specimens, followed by incubation with universal primers that annealed to conserved sequences of the 16S rRNA gene, which is present in all bacteria [41]. The 16S rRNA gene size is approximately 1500 base pairs and comprises nine highly conserved and nine hypervariable regions named V1–V9. The PCR procedure amplifies all or some of the hypervariable and species-specific regions [41]. The conserved regions serve as universal primer binding sites for PCR amplification, whereas the hypervariable regions exhibit sequence diversity and are used for identification purposes. The resulting amplicon is then sequenced, and the results are compared with data deposited in GenBank or other comprehensive genomic databases, or the products are hybridized with species-specific probes. Analysis of large fragments (>1000 bp) may improve species discrimination, but the amplification of shorter fragments (<1000 bp) is more sensitive and results in better-quality sequence data [42]. The hypervariable regions V2 (nucleotides 137–242), V3 (nucleotides 433–497), and V6 (nucleotides 986–1043) contain the maximum nucleotide heterogeneity and, thus, the optimum discriminatory power [43].

The use of the “universal” 16S rRNA gene confers the NAAT’s high sensitivity and specificity and has proven to be particularly useful for microbiological diagnosis in case of the absence of a clinical etiological orientation [41]. The microorganisms-specific primers exhibit superior sensitivity compared to the amplification of universal genes and employ a real-time polymerase chain reaction (rtPCR) platform instead of an agarose gel, enabling the diagnosis to be completed within a few hours [41]. In an in-vitro study in which the performance of PCR primers targeting the 16S rRNA gene and the *rtxA Kingella kingae*-specific operon were compared, the assay targeting the *rtxA* gene had a sensitivity of 30 CFUs/mL, compared to 300 CFUs/mL of the universal gene amplification assay [44]. These results explain the suboptimal performance of the universal primers for detecting the low concentration of *Kingella kingae* organisms usually present in the synovial fluid aspirates (from eleven CFUs/mL to 300 CFUs/mL of synovial fluid, median 15 CFUs/mL) [32].

Alternatively, species-specific tests can be designed to selectively target the most probable bacteria based on epidemiological and clinical considerations (e.g., *Kingella kingae* in toddlers by the *Kingella kingae* Real-Time PCR test, Qualitative, Quest Diagnostic, San Juan Capistrano, California, not yet approved by the FDA) alone or in combination with genes conferring relevant antibiotic-resistance (e.g., *Staphylococcus aureus* and clindamycin-encoding resistance genes in older children [45]). A multiplex panel {BIOFIRE Joint Infection (JI)}, designed to detect bacterial and fungal pathogens commonly associated with adult and pediatric osteoarthritis and pertinent antibiotic-resistance genomic markers, is currently being developed by bioMérieux, Marcy-l’étoile, France [46]. The results of a preliminary evaluation appear promising, although the system has not been approved by the FDA yet. In a study in which its performance was compared to that of conventional cultures, *Kingella kingae* was detected by the novel panel in three of the 112 studied patients. The results were confirmed by isolation of the bacterium, and no false positive results of the molecular method were obtained [46].

The three *Kingella kingae*-specific genes that are targeted by the current NAATs are the *rtxA* and *rtxB* genes that encode the RTXA toxin [44], the *groEL* gene (also known as *cpn60*) that encodes the chaperonin 60 protein [38], and the *mdh* (malate dehydrogenase) gene [47]. Whereas the *groEL*-based assays show suboptimal sensitivity, those that amplify the *rtx* operon do not differentiate between *Kingella kingae* and the *Kingella negevensis* species [47]. El Houmami et al. recently reported a novel real-time assay targeting *Kingella kingae*’s *mdh* gene and challenged it with 18 variants of the gene’s sequence. The test detected seven *Kingella kingae* infections that were missed by PCR tests targeting the *groEL* gene and the *rtx* locus, suggesting that it should be considered the preferable molecular diagnostic assay [47].

The use of NAATs has confirmed the prime role of the bacterium in the causation of pediatric osteoarthritis, already suggested by the BCV culture technique [12,39]. In a Swiss study in which NAATs were routinely used, *Kingella kingae* was detected by positive blood and/or bone and/or joint fluid PCR assays in 64 of 134 (47.8%) children aged 6–48 months [12]. The organism was considered the probable etiology in additional 12 (9.0%) children in whom joint or bone samples were not obtained but had a positive NAAT on an oropharyngeal specimen [12]. It should be pointed out that *Kingella kingae* was not detected among 13 infants younger than six months and was documented in only two of 70 (2.9%) children aged five-15 years [12]. This remarkable age-related morbidity overlaps the epidemiological curve of oropharyngeal colonization by the organism [48]. The pharyngeal carriage of the organism is null in the first life semester and gradually increases to 10–12% among children between the ages of six and 24 months, decreasing after that [48]. This age-related pattern has been attributed to vertical maternal immunity and lack of social contacts in early life, followed by vanishing maternal antibodies and increasing social mingling in toddlers and immunological maturation in older children, resulting in the eradication of colonizing *Kingella kingae* organisms and protection from invasive disease [13].

#### 4.3.1. Detecting Oropharyngeal *Kingella kingae* Colonization as a Diagnostic Tool

Since *Kingella kingae* frequently invades joints or bones that are small and/or not easily accessible, synovial fluid aspirates, bone exudates, or intervertebral disc tissue samples are frequently unavailable for analysis [13,49]. As *Kingella kingae* colonization of the oropharynx is a precondition for invasion of the blood stream and dissemination to bones, joints, and intervertebral discs, an alternate non-invasive diagnostic method based on the detection of mucosal carriage has been developed [50,51,52,53]. The approach consists of obtaining an oropharyngeal specimen and subjecting it to a sensitive *Kingella kingae*-specific molecular assay [50,51,52,53]. The sensitivity and specificity of the test were 100% and 90.5%, respectively, with 95% confidence intervals of 88.4–100% and 82.1–95.8%, respectively. The accuracy was estimated to be 93% (95% confidence intervals of 86.6–96.9%) [51].

A pauci-symptomatic clinical presentation in a young child and normal or mildly elevated inflammation markers, coupled with a positive molecular test, support *Kingella kingae* as the etiology of the disease [49]. This diagnostic strategy has been particularly useful for diagnosing spondylodiscitis cases that otherwise would have remained bacteriologically unconfirmed, and has established *Kingella kingae* as the prime agent of the infection below four years of age [52,53]. The oropharyngeal specimen can also be seeded onto BAV (blood-agar vancomycin) plates [13,54]. This selective medium has been designed to enhance the detection of *Kingella kingae*’s β-hemolytic colonies by inhibiting the growth of Gram-positive bacteria [13,54]. A positive culture result has a diagnostic value similar to that of a NAAT. However, because the sensitivity of the BAV culture is inferior to that of the PCR assay, the test has low negative predictive value. Failure to isolate *Kingella kingae* in an oropharyngeal specimen does not rule out the organism as the agent of a skeletal system infection.

Naturally, this novel strategy has the obvious limitation that the background *Kingella kingae*’s pharyngeal carriage rate is 10%–12% in the young pediatric population and twice as high among daycare center attendees, reducing the predictive value of a positive NAAT result [48,54]. On the other hand, because the colonized oropharyngeal epithelium is the source of the bloodborne spread of the bacterium, the negative predictive value of a sensitive molecular test is high and, therefore, the unsuccessful detection of *Kingella kingae*-specific DNA sequences virtually excludes the bacterium as the cause of the infection.

#### 4.3.2. Do NAATs Make the Isolation of *Kingella kingae* Obsolete?

The use of NAATs has improved the detection of *Kingella kingae* in children with arthritis by a four-fold factor compared to the BCV method and shortened the time-to-detection, and they are increasingly employed to detect this elusive bacterium, potentially neglecting the culture of the organism [13]. Table 1 compares the performance of *Kingella kingae’s* culture- and molecular-based detection methods in biological specimens. Despite the remarkable advantages of the molecular assays, the isolation of the bacterium enables complete characterization of the strain, including determining its antibiotic susceptibility. The potential loss of the capability to isolate *Kingella kingae* could hamper future research to identify the species’ genes responsible for invasiveness and tropism for the skeletal system and endocardial tissues. In-depth study of the genomic content of clinical isolates could reveal virulence factors suitable for being targeted by novel therapeutic strategies and contribute to the development of protective vaccines in the future.

### 4.4. Metagenomic Next-Generation Sequencing (mNGS)

Even when the BCV culture method and sensitive NAATs are employed, the CML fails to identify a pathogen in a large fraction of bone and joint infections [12], indicating that novel diagnostic methods will be needed to improve patient care further.

The emerging mNGS is a novel cell-free DNA technology which is already being employed in oncology, non-invasive antenatal testing, and organ transplant rejection screening, and is increasingly moving to the diagnosis of infectious diseases [55]. Patient samples (usually blood, CSF, or other normally sterile body fluids) are collected using a strict sterile technique. The collection tubes are spun down to separate cell-free DNA, mNGS libraries are prepared and sequenced, and reads identified as of human origin are removed. Any remaining sequence is considered to belong to the putative etiology of the infection and is aligned to a curated pathogen database to identify the organism.

In theory, mNGS can detect any pathogen, and currently, this technology can unambiguously identify > 1400 species, and the turnaround time has been shortened to 1–2 days [56]. The detection and identification can be performed without a priori knowledge of the suspected etiologic agent, and, providing a comprehensive database grounded on single nucleotide polymorphisms is available, a resolution at the subspecies or strain level can be achieved [57]. This unbiased, hypothesis-free strategy is particularly suitable for establishing a microbiological diagnosis in immunocompromised patients and infections in which standard cultures and NAATs have failed to detect the culprit [58].

When mNGS is performed in plasma (known as “liquid biopsy”), the method detects not only pathogens circulating in the bloodstream but also those causing focal infections. A pathogen’s DNA sequences leak from infected sites such as bones, joints, and other tissues to the patient’s blood, dispensing with the need for source sample collection by invasive and costly surgical procedures [59].

mNGS has been extensively used in adult patients with periprosthetic joint infections in which cultures have failed to identify a pathogen [60,61]. However, it should be pointed out that no mNGS-based test has yet received FDA approval [55]. Therefore, reports on the use of mNGS in diagnosing pediatric skeletal system infections, in general, and the specific detection of *Kingella kingae*, in particular, are scarce. Ramchandar performed mNGS on joint and bone samples of 42 children with suspected acute skeletal system infections and compared the results with those obtained by cultures and NAATs [56]. Overall, 26 (61.9%) children had a putative pathogen detected by mNGS vs. 24 (57.1%) detected by the comparators. Three bacteremic patients were also positive for the same bacterium by cultures of the infected site and mNGS. mNGS detected *Kingella kingae* DNA in six synovial fluid aspirates, of which five were also positive by PCR.

In a multicenter study, mNGS performed in plasma with the commercially available Karius test (Redwood City, CA, USA), detected *Kingella kingae* in ten young children (median age: 16.5 months, range: 10–23 months) with spondylodiscitis [59]. The blood cultures were negative in all patients, while the standard-of-care PCR assay yielded the diagnosis in a single child (positive vertebral bone) (*p* = *0.0133* by the two-tailed McNemar’s test). The detection of *Kingella kingae* by mNGS enabled narrow antimicrobial coverage in nine patients and established the diagnosis without biopsy in eight (in one child, a biopsy was performed before the Karius test (Redwood City, CA, USA).

mNGS is a novel and promising versatile tool that is expected to change how infectious diseases are diagnosed drastically. Benefits for patients include avoiding invasive diagnostic procedures and identifying traditional and novel pathogens otherwise undetected by culture or NAAT methods. The mNGS approach, however, has not yet fulfilled all its theoretical potential. The current technology is expensive (from 1000 to 2500 USD per test) and requires costly equipment and technical expertise in molecular biology and bioinformatics that are currently unavailable outside the industrialized world. However, a timely mNGS diagnosis may avoid the performance of less informative tests and costly risky diagnostic procedures and shorten hospital stays. The sensitivity of mNGS remains suboptimal and only comparable, but not superior, to that of NAATs [60], and microorganisms detected by routine microbiological methods can be overlooked by mNGS [56]. Although unbiased testing can be an advantage because it may reveal unexpected pathogens, mNGS provides no information on antimicrobial susceptibility and resistance.

The mNGS technology might occasionally provide spurious test results. DNA contamination may originate in the disrupted and colonized mucosal barriers of oncology and transplant patients at an increased risk for opportunistic infections. The commensal human virome (e.g., herpesviruses), bacterial or viral reads from the sample collection site (e.g., the skin microbiome), and even short human DNA pieces such as fragments of the Y chromosome [62], may also result in the detection of an irrelevant microbial species. These false-positive identification results complicate the management of immunocompromised patients. These limitations indicate that mNGS results should be carefully interpreted in the context of the clinical situation, requiring infectious disease acumen and expertise.

## 5. Conclusions

In recent years, developments in microbiologic detection methods have recognized *Kingella kingae* as the prime etiology of skeletal system infections in early childhood. Due to the predominance of the organism in children aged 6 to 48 months, a sensitive species-specific molecular test should be performed on joint and bone specimens in this age group. If no skeletal system specimen is available, an oropharyngeal specimen should be obtained and subjected to a *Kingella kingae*-specific molecular assay. In infants younger than six months and children older than four years, a nucleic acid amplification test targeting the universal 16S rRNA gene is recommended. Large samples can be inoculated into BCVs to enhance the recovery of this fastidious microorganism and determine the antibiotic susceptibility of the isolate. Further improvements in the sensitivity and specificity of mNGS will likely make this novel method the primary means of diagnosing *Kingella kingae* infections in the future.

## Figures and Tables

**Table 1:** Performance of *Kingella kingae* detection by culture and nucleic acid amplification test sequencing.

		Culture			Nucleic Acid Amplification Tests			
		Normally Sterile Body Fluids (Blood, Aspirates, and Exudates)		Oropharyngeal Specimens	Universal Primers	*Kingella kingae*-Specific Primers		
Features		Solid media	Blood culture vial	BAV medium	16S rRNA	*rtx*	*cpn60*	*mdh*
Sensitivity		±	+	+	++	+++	+++	+++
Specificity		+++	+++	+++	+++	++ ^b^	+++	+++
Time-to-positivity		1–4 days	1–4 days	2–5 days	1–2 days ^a^	hours		
Enables	Antibiotic susceptibility testing	Yes			no			
	Typing	Yes			no			
	Colonization studies	N/A ^c^		Yes	yes			
	Transmission studies	N/A ^c^		Yes	no			
	Outbreak investigation	N/A ^c^		Yes	yes			
	Whole-genome sequencing	Yes			no			
	Study of virulence factors	Yes			no			

^a^: including sequencing of the amplicon; ^b^: does not discriminate between *Kingella kingae* and *Kingella negevensis;* ^c^: not applicable. ±: poor (<5%); +: low (25%); ++: good (75%); +++ (optimal (100%).

## Data Availability

The original data can be found in the publications cited in the review.
